# Extracorporeal Life Support for Pediatric Heart Failure

**DOI:** 10.3389/fped.2016.00115

**Published:** 2016-10-20

**Authors:** Christopher R. Burke, D. Michael McMullan

**Affiliations:** ^1^Division of Cardiac Surgery, Seattle Children’s Hospital, Seattle, WA, USA

**Keywords:** extracorporeal life support, pediatrics, critical care, heart failure, congenital heart disease

## Abstract

Extracorporeal life support (ECLS) represents an essential component in the treatment of the pediatric patient with refractory heart failure. Defined as the use of an extracorporeal system to provide cardiopulmonary support, ECLS provides hemodynamic support to facilitate end-organ recovery and can be used as a salvage therapy during acute cardiorespiratory failure. Support strategies employed in pediatric cardiac patients include bridge to recovery, bridge to therapy, and bridge to transplant. Advances in extracorporeal technology and refinements in patient selection have allowed wider application of this therapy in pediatric heart failure patients.

## Introduction

Based on John Gibbon’s pioneering work on the development of a heart–lung machine for cardiac surgery, Bartlett and his colleagues began using extracorporeal support for prolonged periods of time to support critically ill patients in the 1970s (1). The first pediatric cardiac extracorporeal life support (ECLS) survivor was a young child with postcardiotomy myocardial dysfunction following repair of transposition of the great arteries (2). Early clinical successes led to increased use of ECLS to support children with respiratory and cardiac failure. Data from the Extracorporeal Life Support Organization (ELSO) international registry indicate that ECLS has been used to provide cardiac support for more than 19,000 children (3). Despite significant advances in durable and non-durable ventricular assist devices for adult patients, ECLS remains the most commonly used form of mechanical cardiac and cardiopulmonary support in infants and young children.

## Perioperative Support

Perioperative support following surgical intervention for congenital heart disease remains the most common indication (74%) for pediatric cardiac ECLS, followed by support for cardiomyopathy (4.4%), myocarditis (2.6%), cardiac arrest (2.5%), and cardiogenic shock (1.8%) (3). Not surprisingly, venoarterial ECLS is the most common mode of support for these patients (95%) (4). Overall survival in this patient population has increased moderately during the past several decades from 38% (1990–2000) to 45% (2001–2011) but remains less than survival in patients of similar age who require ECLS for respiratory support alone (5). Pediatric cardiac ECLS survival appears to be improving, despite the fact that ECLS is being used to support an increasingly heterogeneous patient population. Single-ventricle heart disease, once considered a contraindication for ECLS, now represents one of the most common diagnoses in neonates who require extracorporeal support (6). Increased utilization has also been observed in other unique patient populations during the past decade, including ECLS for refractory cardiac arrest (ECPR, over 4,000 cases in the ELSO registry) and patients being bridged to transplantation (284 cases reported in the ELSO registry) (3, 4).

Despite advances in perioperative care, myocardial protection, anesthesia, and surgical techniques, children remain at risk for myocardial dysfunction following heart surgery. In most cases, myocardial dysfunction is mild and improves with inotropic therapy, afterload reduction, and pulmonary vasodilators. In its most severe form, postoperative myocardial dysfunction may lead to inadequate end-organ oxygen delivery and inability to separate from cardiopulmonary bypass. ECLS may be used as short-term supportive therapy to improve end-organ oxygen delivery, reverse acidosis and other metabolic derangements, prevent progression to cardiac arrest, prevent dysrhythmias, and limit further myocardial injury by decreasing myocardial oxygen demand. Initiation of ECLS provides short-term mechanical circulatory support that enables weaning of inotropic and vasopressor support and facilitates myocardial recovery. The incidence of postoperative ECLS varies by patient population and center, with some centers reporting its use in fewer than 0.6% of patients and others reporting that up to 6.8% of patients require postoperative mechanical support (7, 8). Postoperative ECLS is utilized in as many as 10% of patients who undergo some high-risk procedures, such as surgical palliation of single-ventricle heart disease (9). Overall survival in children who require ECLS for failure to separate from cardiopulmonary bypass is around 45% (10).

Initiation of ECLS for failure to separate from cardiopulmonary bypass should prompt investigation for residual hemodynamically significant lesions. Diagnostic imaging, such as echocardiography or catheterization, should be undertaken promptly when questions remain about adequacy of surgical repair or palliation, or if a patient makes poor clinical progression toward separation from ECLS. Unsuspected lesions may have been found in over half of pediatric ECLS patients who undergo perioperative diagnostic cardiac catheterization (11). In many cases, clinically important residual lesions may be addressed by percutaneous interventional techniques, whereas others may require surgical intervention to facilitate separation from ECLS. Left ventricular distention must be avoided in patients who require postoperative venoarterial ECLS. Ventricular distention increases myocardial wall tension and reduces myocardial oxygen delivery, which has deleterious effects on ventricular recovery and function. In patients with severely depressed left ventricular systolic function, consideration should be given to inserting a left heart vent at time of conversion from cardiopulmonary bypass to ECLS. Effective left ventricle decompression may be accomplished by venting through pulmonary vein, left atrium, or left ventricle apex cannulae. Alternatively, surgical or percutaneous balloon atrial septostomy may be used to adequately decompress the left ventricle during ECLS. Meticulous hemostasis prior to leaving the operating room is essential to reduce the likelihood of postoperative bleeding and infection, which occur in up to 30 and 11%, respectively. Positive routine surveillance mediastinal cultures obtained at time of postoperative surgical interventions, including delayed sternal closure, have been shown to be predictive of postoperative surgical site infection in children (12). Routine ECLS circuit cultures may also detect occult but clinically important infection in pediatric patients who require postoperative ECLS.

Indications and thresholds for initiating ECLS during the perioperative period are not well established. There is evidence that patients who successfully separate from cardiopulmonary bypass but subsequently require ECLS during the postoperative period are more likely to survive than patients who are unable to separate form cardiopulmonary bypass (69 vs. 24% survival) (13). However, there is also evidence that initiation of ECLS in the operating room is associated with improved survival when compared to initiation of support in the intensive care unit (64 vs. 29%) (14). Irrespective of indication for and location of support, prolonged duration of ECLS is an independent risk factor for death. ECLS survival is typically >50% when support is limited to <72 h but is <40% for patients supported >72 h (15, 16). Survival following 7 days of ECLS support is very low in this population (17).

## Bridge to Transplant

Extracorporeal life support may be used to support children with severe, life-threatening congenital and non-congenital forms of heart failure. The most common forms of non-congenital heart disease in this population are cardiomyopathy (16% of pediatric cardiac ECLS cases) and myocarditis (8% of cases) (4). In general, survival is better in these patients (62% cardiomyopathy; 70% myocarditis) than in those with structural congenital etiologies of heart failure because in many cases, the underlying disease process is self-limited or manageable with appropriate pharmacotherapy. Additional causes of pediatric heart failure that may require ECLS include refractory or chronic dysrhythmia, infection, and myocardial infarction (18, 19).

Despite increased risk associated with prolonged support, ECLS has been used to provide mechanical cardiac support for children awaiting heart transplantation for nearly 30 years (20–24). The ELSO registry contains data from over 280 pediatric patients who were successful bridged to cardiac transplantation using ECLS (4). Single-center reports of pediatric patients who receive ECLS while awaiting heart transplantation suggest that up to 24% of patients may recover adequate myocardial function to be weaned from ECLS prior to transplantation and that approximately 50% of patients are successfully transplanted (23, 24). Early posttransplant survival is approximately 80%, and survival in patients supported with ECLS appears to be similar to that in patients who do not requite extracorporeal bridge to transplantation at 1 year.

Retrospective analysis of ELSO and Organ Procurement Transplant Network data suggest that up to 45% of children awaiting heart transplantation while receiving ECLS may ultimately receive a donor organ, but only 47% of these patients survive to hospital discharge (25). In contrast, bridge to transplantation with the Berlin Heart EXCOR, the only pediatric ventricular assist device approved for use in the United States, is successful in 64% of patients (26). Approximately 75% of these patients remain alive at 12 months but roughly one-third of children supported with the Berlin Heart device experience clinically significant neurologic injury prior to transplantation (26). It is clear that ECLS and currently available ventricular assist devices are inferior to adult mechanical bridge to transplant options. Despite this, ECLS will likely continue to play a crucial role in stabilization of critically ill young children and, in many cases, will serve as a viable option for bridge-to-bridge or bridge-to-decision (27, 28). In addition, ECLS is probably the best mode of mechanical cardiac support for infants and young children with single-ventricle heart disease and refractory heart failure.

## Extracorporeal Cardiopulmonary Resuscitation

Perhaps, the most significant advancement in the field of pediatric ECLS during the last several decades is the use of ECLS as an adjunct to standard cardiopulmonary resuscitation (ECPR). The earliest reported use of ECPR was in 1976 (29). Since that time, a number of advances in ECLS equipment, refinements in surgical techniques, and growing recognition of an apparent survival benefit have led to increased use of ECPR in pediatric patients. Hospitals worldwide have developed rapid-response ECPR programs to expeditiously initiate ECLS during cardiopulmonary resuscitation, often within 30 min of cardiac arrest (30). To date, over 6,400 ECPR cases have been reported to the ELSO registry, with an overall survival rate of 36.8% (3). Despite the absence of randomized, controlled trials to support the use of ECPR, there is strong evidence that ECPR provides a significant survival advantage over conventional cardiopulmonary resuscitation in children (37 vs. 13–27%) (31). Overall survival following ECPR appears to be higher for neonates (41%) and children (41%) than adults (30%), which may reflect age-related differences in etiology of cardiac arrest and patient comorbidities (3). ECPR survival is greater in children with two functional ventricles than in those who have single-ventricle congenital heart disease (32, 33). Survival is as high as 89% in children with cardiomyopathy who experience cardiac arrest (33). Overall ECPR survival is lower (21%) and central nervous system hemorrhage is higher in neonates born before 34-week gestational age than it is for all preterm infants (27% survival) (34).

There is no consensus regarding the impact of duration of pre-ECLS cardiopulmonary resuscitation on survival in children. Some retrospective studies suggest that duration of pre-ECPR resuscitation impacts survival (31). Although survival following conventional resuscitation efforts >30 min is rare, survival is not uncommon in patients who receive ECPR following 45–60 min of conventional resuscitation (30, 31, 35, 36), and the use of conventional resuscitative measures for >30 min before initiation of ECPR is not necessarily associated with poorer outcome (30). The impact of duration of pre-ECPR resuscitation on survival may vary by age and patient population. Increased duration of pre-ECPR resuscitation has been shown to be associated with worse outcome in adults, using propensity score-matched analysis (36). Adequacy, rather than duration, of pre-ECPR resuscitation is undoubtedly the most important determinant of ECPR outcomes (37–39).

Not surprisingly, patients who require ECPR are at risk for significant ECLS and resuscitation-related complications. Clinically significant hemorrhage is seen in as many as 30% of patients (32). Acute kidney injury that requires renal replacement therapy occurs in 8.7% and bloodstream infections in 8.5% of patients (32). Central nervous system injury occurs in approximately 11% of ECPR patients and is independently associated with increased mortality (34). Approximately 80% of pediatric ECPR survivors have a Pediatric Cerebral Performance Category score ≤2 at hospital discharge or follow-up, indicating mild or no neurological disability (30). Information about long-term neurologic outcome is limited, but a recent study reported that children who survived ECPR scored 76.5% on full-scale intelligence quotient testing 5 years after hospital discharge (40).

Despite increasing use by a growing number of centers, ECPR remains a relatively infrequently utilized therapy, representing only 8% of ECLS cases reported to the ELSO registry (3). When properly executed, ECPR requires expeditious mobilization of a large number of hospital resources and coordinated interaction between several interprofessional teams. Many centers now perform ECPR simulation training to increase team preparedness and reduce time to ECLS initiation. ECLR simulation has been found to improve practitioner confidence and knowledge regarding deployment of ECPR (41, 42) and reduce ECLS initiation times during ECPR events (43). ECLS simulation is recognized as a critical component in the development and maintenance of successful ECPR programs.

## Contraindications

Pediatric cardiac ECLS is performed in a heterogeneous patient population that in many cases would not survive without extracorporeal support. Therefore, it is difficult to determine absolute contraindications for its use. In general, potential ECLS candidates must be free of a lethal cardiac condition not amenable to surgery or any other terminal condition, including a severe and irreversible brain injury. The ECLS team must assess each candidate for bleeding risk, and those with severe bleeding diatheses are generally not offered ECLS. Historically, extreme prematurity and low weight have been considered contraindications; however, determining absolute cutoffs for gestational age or weight has proven difficult. Extrapolating data from neonatal respiratory ECLS, Smith et al. showed that neonates with gestational age <32 weeks had a 38% rate of CNS hemorrhage (44). In this report of ELSO data, there was a stepwise decrease in mortality as gestational age increased. Furthermore, McMullan et al. showed that survival following neonatal ECPR decreased with younger gestational age (34).

Several reports have addressed the association between patient weight and pediatric cardiac ECLS outcome. For example, infants placed on ECLS weighing <2.5 kg with hypoplastic left heart syndrome have been found to have a survival of only 10% (45). A follow-up study examined outcomes in neonates <3 kg placed on ECLS following cardiac surgery (46). In this series, 30-day survival was 33%. Finally, neonates <2 kg that underwent ECPR were found to have a greater than four times risk of mortality as compared to newborns >3 kg (34). The ELSO registry contains very little data on patients at the extreme lower end of gestational age or weight, making the determination of absolute cutoff values for gestational age or weight impossible. Therefore, clinicians must balance risks and potential benefits on an individual patient basis, understanding the risk of neurologic injury and death increases in smaller and younger infants.

## Complications

### Hemorrhagic

Children who require ECLS are at risk for a number of clinically significant and life-threatening complications. Postoperative hemorrhage is an important risk factor for mortality in children who require ECLS during the postoperative period (47). Hemorrhagic complications, including surgical and cannulation site bleeding, occur in >30% of patient populations, such as patients who require perioperative support (3). Cardiopulmonary bypass causes platelet dysfunction due to contact activation and consumption of platelets and clotting factors, which increases the risk of hemorrhage (48). In addition, cardiopulmonary bypass-induced hemodilution further increases the risk of postoperative bleeding (49). ECLS circuit surface coatings are frequently used to reduce the risk of circuit-related derangements in the coagulation system, but there is little clinical evidence to support the use of surface coatings or for the superiority of one coating over another. Immaturity of the coagulation cascade makes neonates particularly vulnerable to ECLS-related hemorrhage during the postoperative period (50). The impact of ECLS-related bleeding on survival appears to vary by age, with neonates exhibiting worse overall survival (31%) than older children (50%). Patients at increased risk for bleeding may be safely managed with reduced or delayed systemic anticoagulation for a limited period of time (51). In cases of refractory hemorrhage, recombinant Factor VII can been used to effectively control bleeding in many patients, albeit with a 19–24% rate of thromboembolic complications (52–55).

### CNS Injury

Central nervous injury is also a relatively common and devastating complication for pediatric cardiac ECLS patients. CNS injury represents a heterogenous group of disorders that include CNS hemorrhage, ischemic stroke, seizures, and anoxic brain injury. Neonates, especially premature infants, are at greatest risk (11%) for experiencing central nervous system hemorrhage (3). The need for anticoagulation, along with the presence of an arterial cannula (during venoarterial ECLS), places these patients at increased risk for both thrombotic and hemorrhagic CNS complications. Other risk factors for severe CNS injury in pediatric ECLS patients include metabolic acidosis, inotropic/vasopressor medication usage, renal failure, cardiopulmonary resuscitation, or LVAD placement prior to ECLS initiation (56). Pediatric cardiac ECLS patients have also been found to have higher rates of CNS hemorrhage as compared to medical patients, and CNS hemorrhage has been shown to significantly increase mortality (47). A recent ELSO report reviewed cannulation trends and neurologic complications and found that pediatric patients placed on venoarterial ECLS have been found to be 1.6 times more likely to suffer CNS injury than those placed on venovenous support; interestingly, no difference was observed in rates of neurologic complications between carotid and femoral cannulation among venoarterial patients (57). Seizures are a relatively common manifestation of CNS injury in pediatric ECLS patients and portend a poor outcome. Seizure activity can be difficult to detect in these patients, as most are sedate and/or chemically paralyzed. However, the utility of routine use of monitoring to detect seizure activity, using electroencephalograms (EEGs) or amplitude-integrated EEG, remains controversial and an area of active investigation (58, 59).

### Other Complications

Additional complications include oxygenator failure, pericardial tamponade, pneumothorax, acidosis, seizures, infection, renal failure, and limb ischemia. Renal failure is an independent predictor of mortality in several ECLS patient populations (32, 34). Acute renal failure (GFR <35 ml/min/1.73 m) is relatively common and has been reported in up to 72% of pediatric cardiac ECLS patients with 59% requiring continuous renal replacement therapy (CRRT) (60). Risk factors for renal failure in pediatric ECLS patients include increased duration of extracorporeal support, hemolysis, increased pre-ECLS creatinine level, and pre-ECLS acidosis (60, 61). Severity of pre-ECLS acidemia is associated with reduced survival in neonates who require ECLS, suggesting that earlier initiation of ECLS may reduce the degree and duration of inadequate oxygen delivery and improve survival in these patients (62).

## Summary

The field of pediatric ECLS has experienced remarkable technological and medical advances that have led to wider acceptance and broader applications of this lifesaving therapy during recent decades. The origins of contemporary ECLS are based on the pioneering work of John Gibbon in 1931, when he wrote “*the idea naturally occurred to me that if it were possible to remove continuously some of the blue blood from the patient’s swollen veins, put oxygen into that blood and allow carbon dioxide to escape from it, and then inject continuously the now-red blood into the patient’s arteries, we might have saved her life*” (2). Nearly 90 years later, his vision has been realized. Continuous improvements in circuit design, individualized anticoagulation strategies, and recognition of the importance of ECLS in the management of pediatric heart failure will undoubtedly lead to increased use of this highly complex but efficacious support therapy.

## Author Contributions

CB – conception of manuscript, literature review, manuscript preparation, and final revisions. DM – conception of manuscript, critical manuscript revisions, and final revisions.

## Conflict of Interest Statement

The authors declare that the research was conducted in the absence of any commercial or financial relationships that could be construed as a potential conflict of interest.
